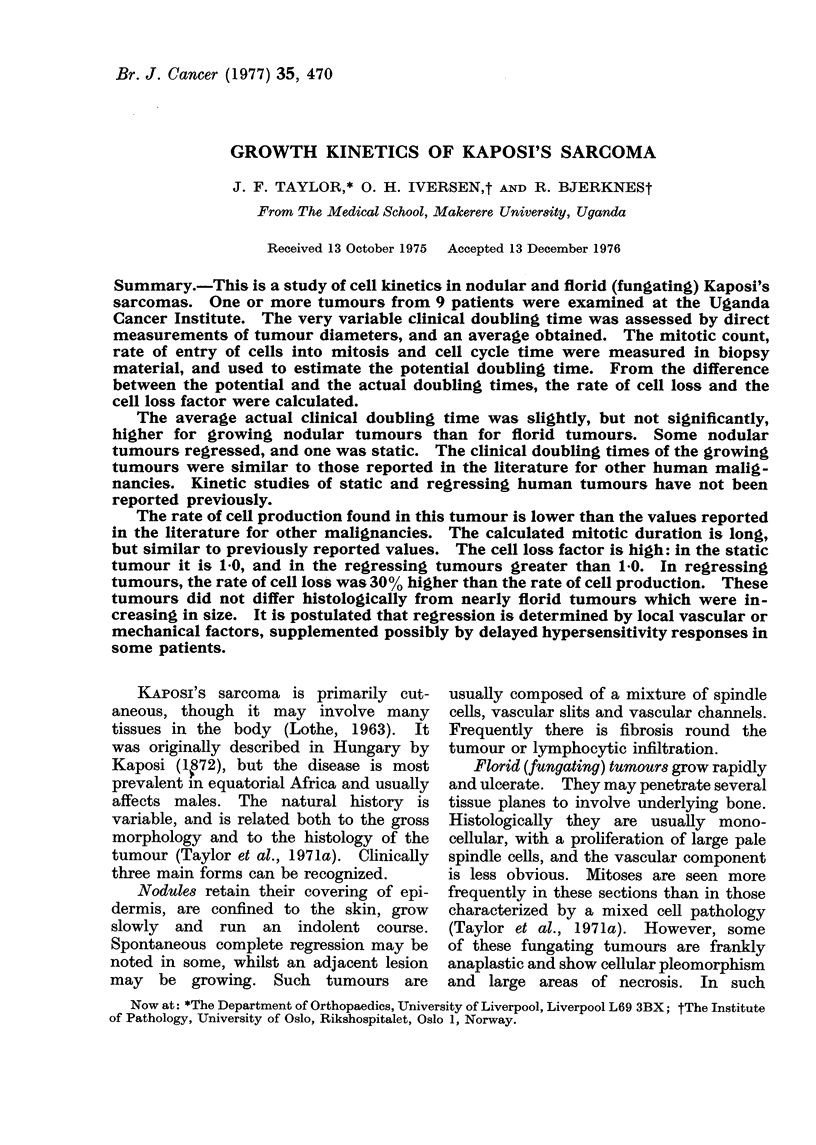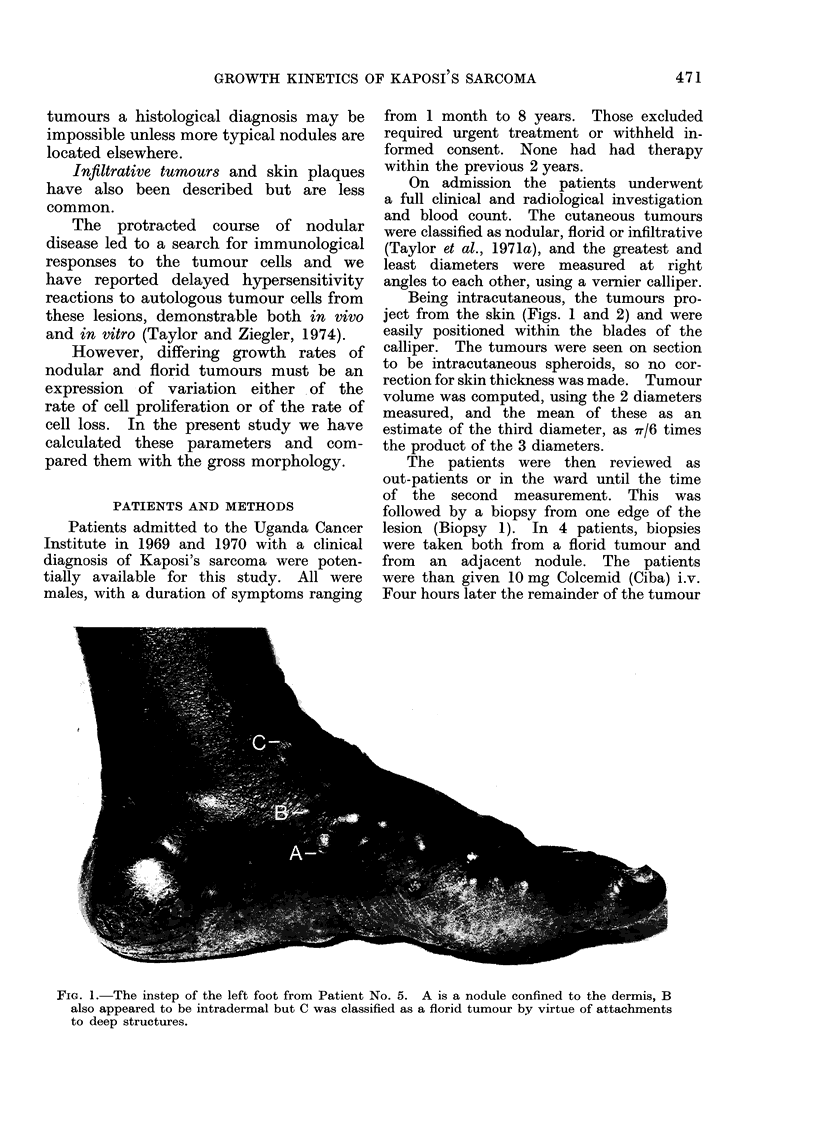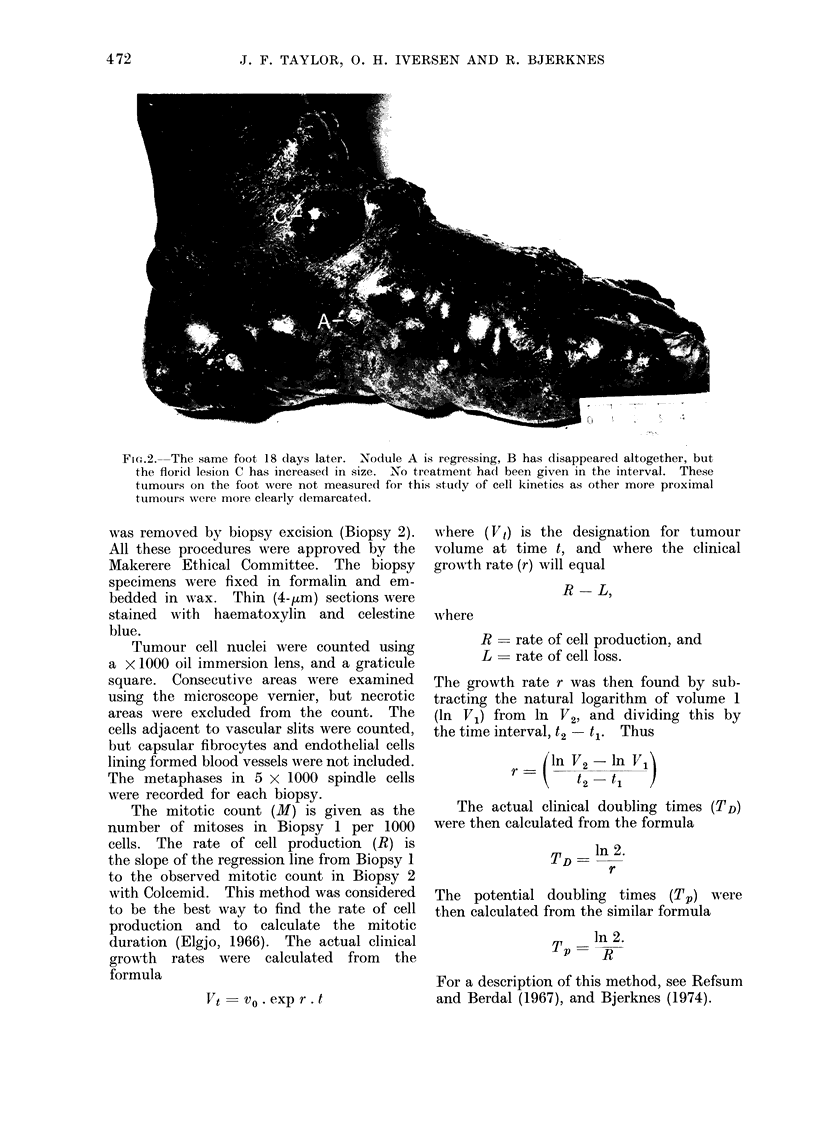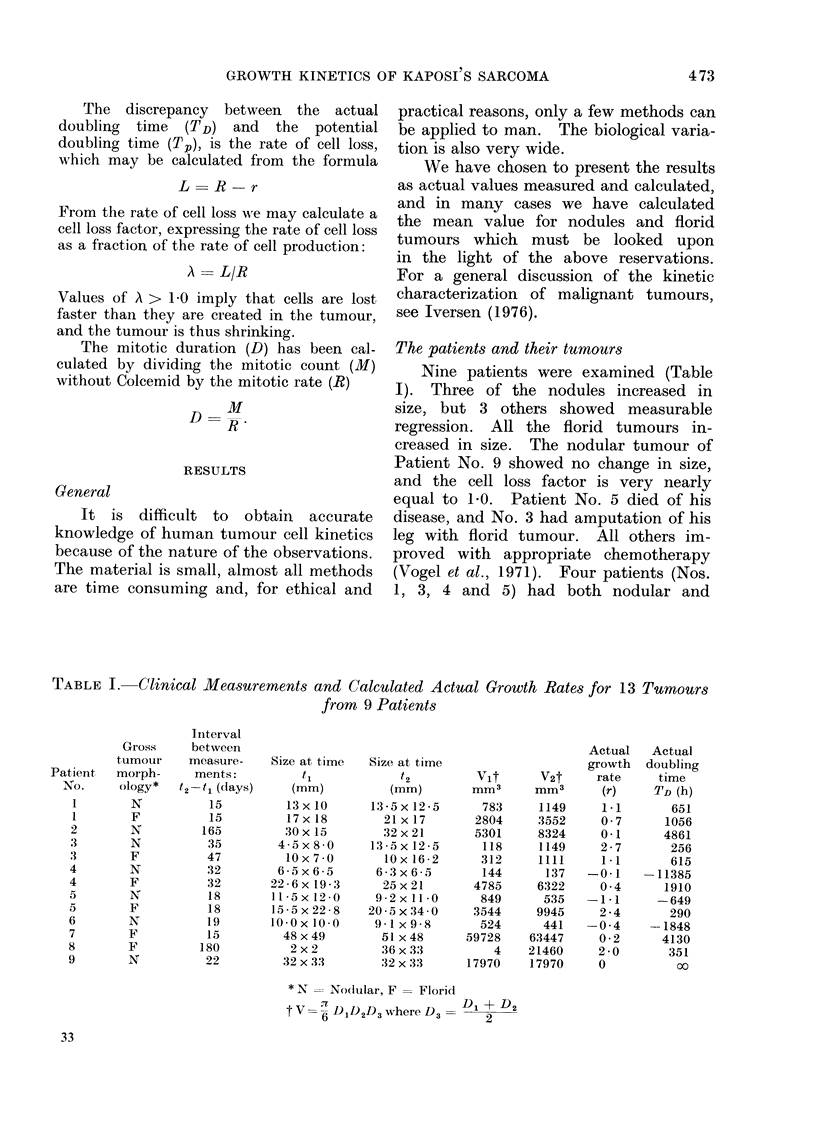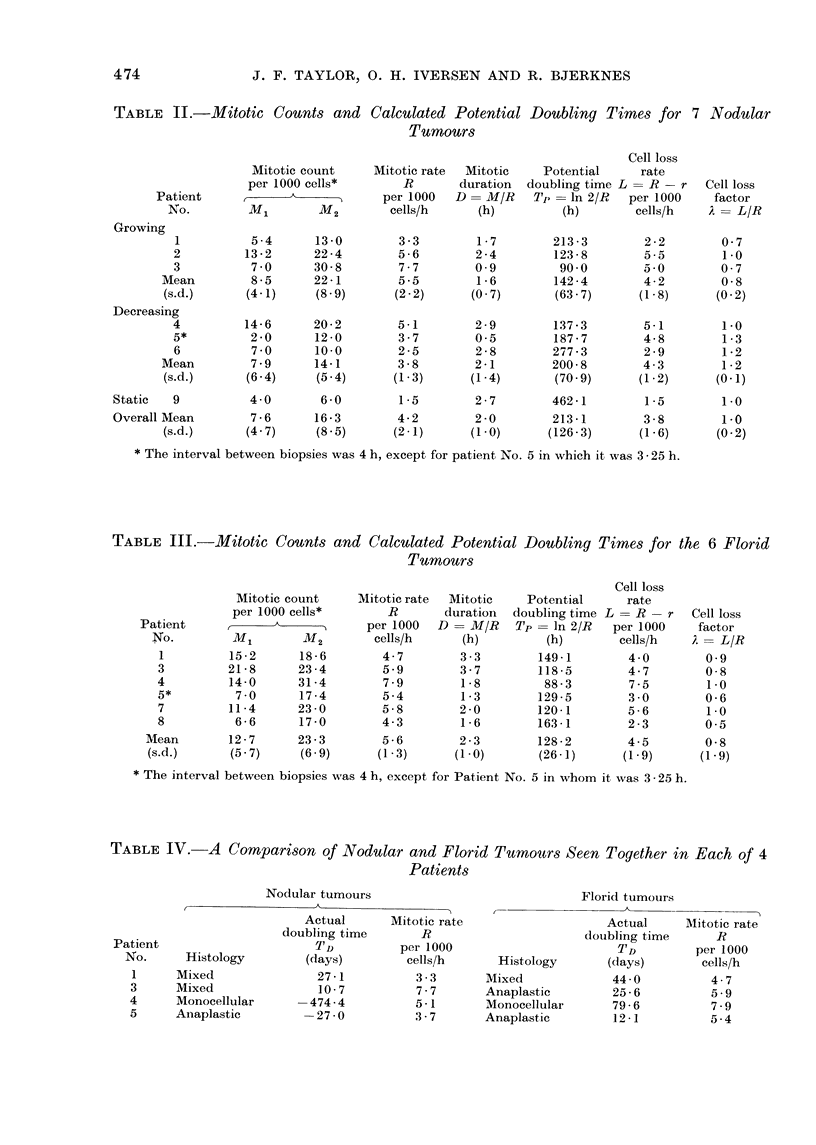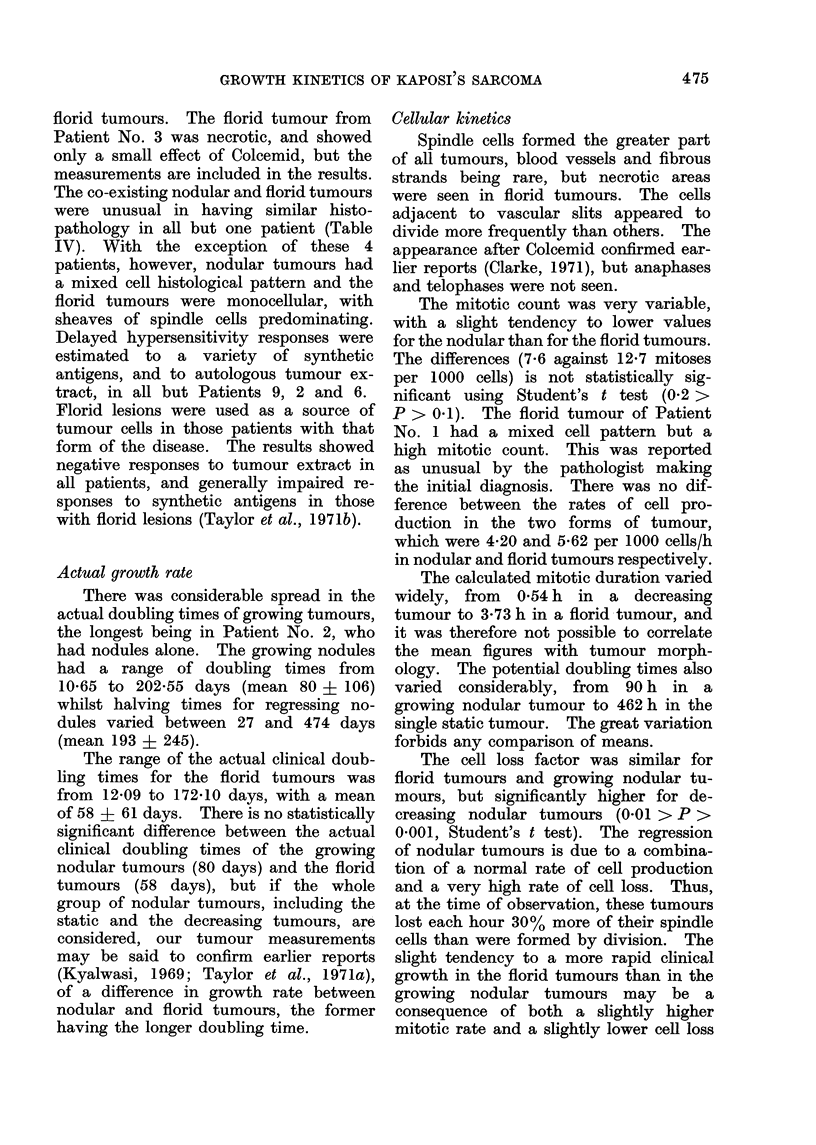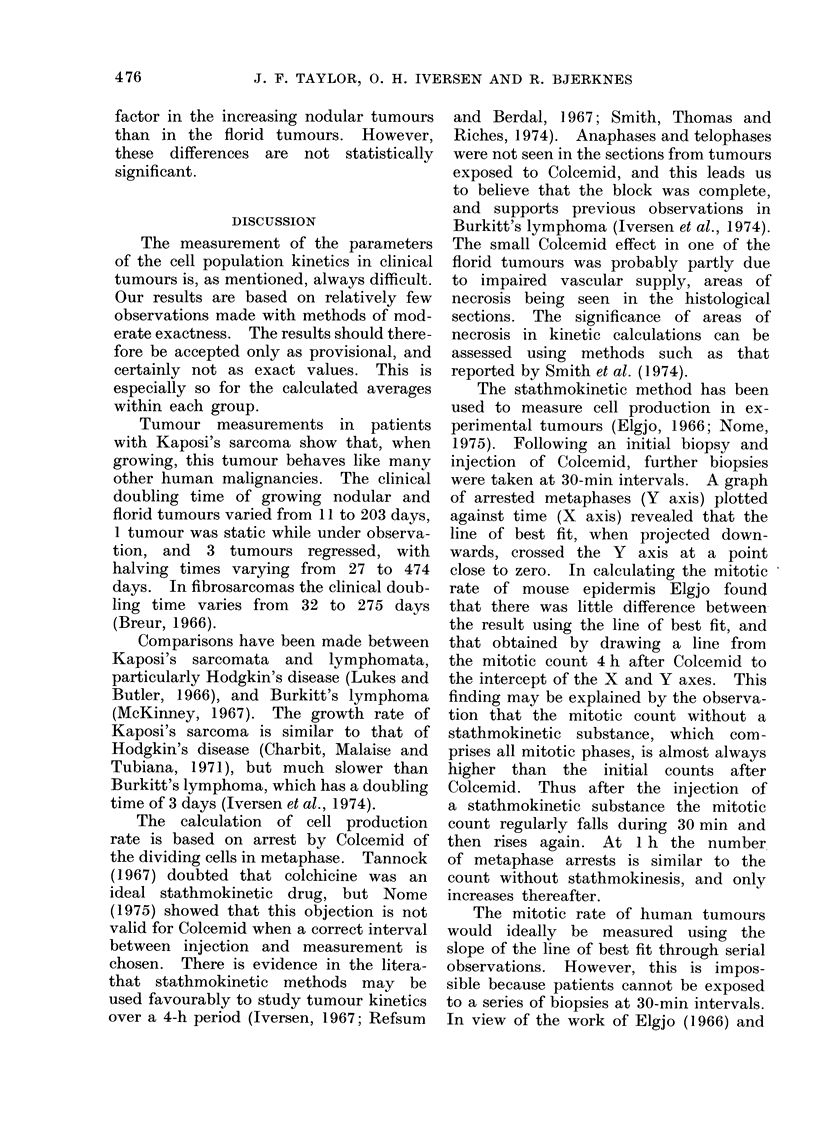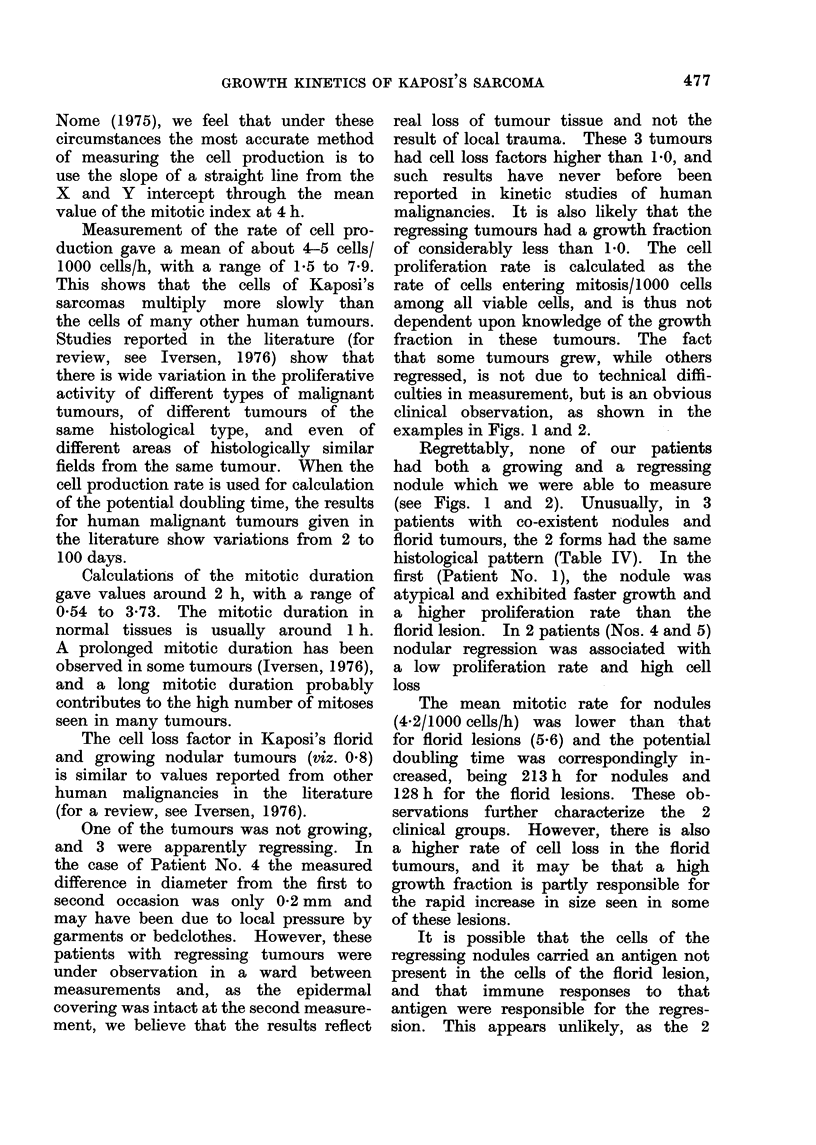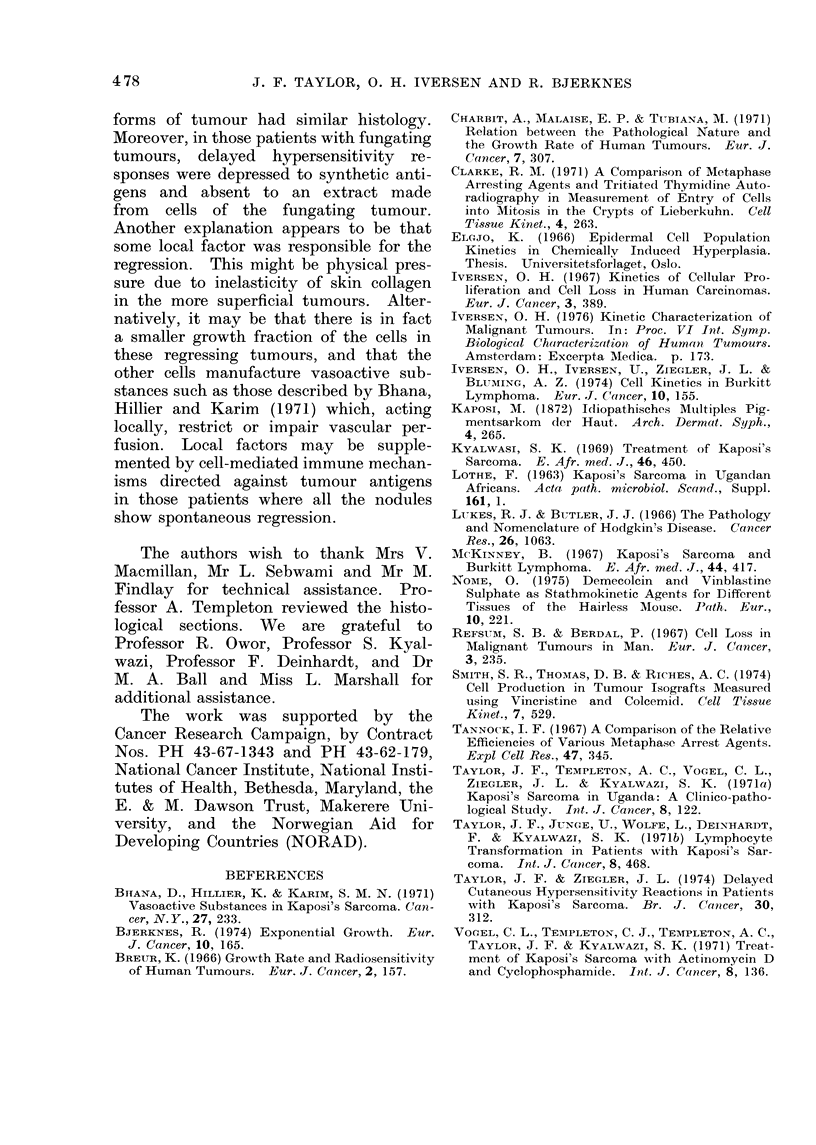# Growth kinetics of Kaposi's sacroma.

**DOI:** 10.1038/bjc.1977.70

**Published:** 1977-04

**Authors:** J. F. Taylor, O. H. Iversen, R. Bjerknes

## Abstract

**Images:**


*Br. J. Cancer* (1977) **35**, 470

### GROWTH KINETICS OF KAPOSI'S SARCOMA

J. F. TAYLOR,* O. H. IVERSEN,† AND R. BJERKNES†

*From The Medical School, Makerere University, Uganda*

Received 13 October 1975 Accepted 13 December 1976

**Summary.—This is a study of cell kinetics in nodular and florid (fungating) Kaposi's sarcomas. One or more tumours from 9 patients were examined at the Uganda Cancer Institute. The very variable clinical doubling time was assessed by direct measurements of tumour diameters, and an average obtained. The mitotic count, rate of entry of cells into mitosis and cell cycle time were measured in biopsy material, and used to estimate the potential doubling time. From the difference between the potential and the actual doubling times, the rate of cell loss and the cell loss factor were calculated.**

**The average actual clinical doubling time was slightly, but not significantly, higher for growing nodular tumours than for florid tumours. Some nodular tumours regressed, and one was static. The clinical doubling times of the growing tumours were similar to those reported in the literature for other human malignancies. Kinetic studies of static and regressing human tumours have not been reported previously.**

**The rate of cell production found in this tumour is lower than the values reported in the literature for other malignancies. The calculated mitotic duration is long, but similar to previously reported values. The cell loss factor is high: in the static tumour it is 1·0, and in the regressing tumours greater than 1·0. In regressing tumours, the rate of cell loss was 30% higher than the rate of cell production. These tumours did not differ histologically from nearly florid tumours which were increasing in size. It is postulated that regression is determined by local vascular or mechanical factors, supplemented possibly by delayed hypersensitivity responses in some patients.**

KAPOSI'S sarcoma is primarily cutaneous, though it may involve many tissues in the body (Lothe, 1963). It was originally described in Hungary by Kaposi (1872), but the disease is most prevalent in equatorial Africa and usually affects males. The natural history is variable, and is related both to the gross morphology and to the histology of the tumour (Taylor *et al.*, 1971*a*). Clinically three main forms can be recognized.

*Nodules* retain their covering of epidermis, are confined to the skin, grow slowly and run an indolent course. Spontaneous complete regression may be noted in some, whilst an adjacent lesion may be growing. Such tumours are usually composed of a mixture of spindle cells, vascular slits and vascular channels. Frequently there is fibrosis round the tumour or lymphocytic infiltration.

*Florid* (*fungating*) *tumours* grow rapidly and ulcerate. They may penetrate several tissue planes to involve underlying bone. Histologically they are usually monocellular, with a proliferation of large pale spindle cells, and the vascular component is less obvious. Mitoses are seen more frequently in these sections than in those characterized by a mixed cell pathology (Taylor *et al.*, 1971*a*). However, some of these fungating tumours are frankly anaplastic and show cellular pleomorphism and large areas of necrosis. In such

Now at: *The Department of Orthopaedics, University of Liverpool, Liverpool L69 3BX; †The Institute of Pathology, University of Oslo, Rikshospitalet, Oslo 1, Norway.

tumours a histological diagnosis may be impossible unless more typical nodules are located elsewhere.

*Infiltrative tumours* and skin plaques have also been described but are less common.

The protracted course of nodular disease led to a search for immunological responses to the tumour cells and we have reported delayed hypersensitivity reactions to autologous tumour cells from these lesions, demonstrable both *in vivo* and *in vitro* (Taylor and Ziegler, 1974).

However, differing growth rates of nodular and florid tumours must be an expression of variation either of the rate of cell proliferation or of the rate of cell loss. In the present study we have calculated these parameters and compared them with the gross morphology.

### PATIENTS AND METHODS

Patients admitted to the Uganda Cancer Institute in 1969 and 1970 with a clinical diagnosis of Kaposi's sarcoma were potentially available for this study. All were males, with a duration of symptoms ranging from 1 month to 8 years. Those excluded required urgent treatment or withheld informed consent. None had had therapy within the previous 2 years.

On admission the patients underwent a full clinical and radiological investigation and blood count. The cutaneous tumours were classified as nodular, florid or infiltrative (Taylor *et al.*, 1971*a*), and the greatest and least diameters were measured at right angles to each other, using a vernier calliper.

Being intracutaneous, the tumours project from the skin (Figs. 1 and 2) and were easily positioned within the blades of the calliper. The tumours were seen on section to be intracutaneous spheroids, so no correction for skin thickness was made. Tumour volume was computed, using the 2 diameters measured, and the mean of these as an estimate of the third diameter, as $\pi/6$ times the product of the 3 diameters.

The patients were then reviewed as out-patients or in the ward until the time of the second measurement. This was followed by a biopsy from one edge of the lesion (Biopsy 1). In 4 patients, biopsies were taken both from a florid tumour and from an adjacent nodule. The patients were than given 10 mg Colcemid (Ciba) i.v. Four hours later the remainder of the tumour

FIG. 1.—The instep of the left foot from Patient No. 5. A is a nodule confined to the dermis, B also appeared to be intradermal but C was classified as a florid tumour by virtue of attachments to deep structures.

FIG. 2.—The same foot 18 days later. Nodule A is regressing, B has disappeared altogether, but the florid lesion C has increased in size. No treatment had been given in the interval. These tumours on the foot were not measured for this study of cell kinetics as other more proximal tumours were more clearly demarcated.

was removed by biopsy excision (Biopsy 2). All these procedures were approved by the Makerere Ethical Committee. The biopsy specimens were fixed in formalin and embedded in wax. Thin (4-$\mu$m) sections were stained with haematoxylin and celestine blue.

Tumour cell nuclei were counted using a $\times 1000$ oil immersion lens, and a graticule square. Consecutive areas were examined using the microscope vernier, but necrotic areas were excluded from the count. The cells adjacent to vascular slits were counted, but capsular fibrocytes and endothelial cells lining formed blood vessels were not included. The metaphases in $5 \times 1000$ spindle cells were recorded for each biopsy.

The mitotic count ($M$) is given as the number of mitoses in Biopsy 1 per 1000 cells. The rate of cell production ($R$) is the slope of the regression line from Biopsy 1 to the observed mitotic count in Biopsy 2 with Colcemid. This method was considered to be the best way to find the rate of cell production and to calculate the mitotic duration (Elgjo, 1966). The actual clinical growth rates were calculated from the formula

$$V_t = v_0 \,.\, \exp r \,.\, t$$

where ($V_t$) is the designation for tumour volume at time $t$, and where the clinical growth rate ($r$) will equal

$$R - L,$$

where

$R$ = rate of cell production, and
$L$ = rate of cell loss.

The growth rate $r$ was then found by subtracting the natural logarithm of volume 1 ($\ln V_1$) from $\ln V_2$, and dividing this by the time interval, $t_2 - t_1$. Thus

$$r = \left(\frac{\ln V_2 - \ln V_1}{t_2 - t_1}\right)$$

The actual clinical doubling times ($T_D$) were then calculated from the formula

$$T_D = \frac{\ln 2.}{r}$$

The potential doubling times ($T_p$) were then calculated from the similar formula

$$T_p = \frac{\ln 2.}{R}$$

For a description of this method, see Refsum and Berdal (1967), and Bjerknes (1974).

The discrepancy between the actual doubling time ($T_D$) and the potential doubling time ($T_p$), is the rate of cell loss, which may be calculated from the formula

$$L = R - r$$

From the rate of cell loss we may calculate a cell loss factor, expressing the rate of cell loss as a fraction of the rate of cell production:

$$\lambda = L/R$$

Values of $\lambda > 1{\cdot}0$ imply that cells are lost faster than they are created in the tumour, and the tumour is thus shrinking.

The mitotic duration ($D$) has been calculated by dividing the mitotic count ($M$) without Colcemid by the mitotic rate ($R$)

$$D = \frac{M}{R}.$$

### RESULTS

### *General*

It is difficult to obtain accurate knowledge of human tumour cell kinetics because of the nature of the observations. The material is small, almost all methods are time consuming and, for ethical and practical reasons, only a few methods can be applied to man. The biological variation is also very wide.

We have chosen to present the results as actual values measured and calculated, and in many cases we have calculated the mean value for nodules and florid tumours which must be looked upon in the light of the above reservations. For a general discussion of the kinetic characterization of malignant tumours, see Iversen (1976).

### *The patients and their tumours*

Nine patients were examined (Table I). Three of the nodules increased in size, but 3 others showed measurable regression. All the florid tumours increased in size. The nodular tumour of Patient No. 9 showed no change in size, and the cell loss factor is very nearly equal to 1·0. Patient No. 5 died of his disease, and No. 3 had amputation of his leg with florid tumour. All others improved with appropriate chemotherapy (Vogel *et al.*, 1971). Four patients (Nos. 1, 3, 4 and 5) had both nodular and

TABLE I.—*Clinical Measurements and Calculated Actual Growth Rates for* 13 *Tumours from* 9 *Patients*

| Patient No. | Gross tumour morphology* | Interval between measurements: $t_2 - t_1$ (days) | Size at time $t_1$ (mm) | Size at time $t_2$ (mm) | $V_1$† $mm^3$ | $V_2$† $mm^3$ | Actual growth rate ($r$) | Actual doubling time $T_D$ (h) |
|---|---|---|---|---|---|---|---|---|
| 1 | N | 15 | 13 × 10 | 13·5 × 12·5 | 783 | 1149 | 1·1 | 651 |
| 1 | F | 15 | 17 × 18 | 21 × 17 | 2804 | 3552 | 0·7 | 1056 |
| 2 | N | 165 | 30 × 15 | 32 × 21 | 5301 | 8324 | 0·1 | 4861 |
| 3 | N | 35 | 4·5 × 8·0 | 13·5 × 12·5 | 118 | 1149 | 2·7 | 256 |
| 3 | F | 47 | 10 × 7·0 | 10 × 16·2 | 312 | 1111 | 1·1 | 615 |
| 4 | N | 32 | 6·5 × 6·5 | 6·3 × 6·5 | 144 | 137 | −0·1 | −11385 |
| 4 | F | 32 | 22·6 × 19·3 | 25 × 21 | 4785 | 6322 | 0·4 | 1910 |
| 5 | N | 18 | 11·5 × 12·0 | 9·2 × 11·0 | 849 | 535 | −1·1 | −649 |
| 5 | F | 18 | 15·5 × 22·8 | 20·5 × 34·0 | 3544 | 9945 | 2·4 | 290 |
| 6 | N | 19 | 10·0 × 10·0 | 9·1 × 9·8 | 524 | 441 | −0·4 | −1848 |
| 7 | F | 15 | 48 × 49 | 51 × 48 | 59728 | 63447 | 0·2 | 4130 |
| 8 | F | 180 | 2 × 2 | 36 × 33 | 4 | 21460 | 2·0 | 351 |
| 9 | N | 22 | 32 × 33 | 32 × 33 | 17970 | 17970 | 0 | ∞ |

* N = Nodular, F = Florid

† $V = \frac{\pi}{6} D_1 D_2 D_3$ where $D_3 = \frac{D_1 + D_2}{2}$

TABLE II.—*Mitotic Counts and Calculated Potential Doubling Times for 7 Nodular Tumours*

| Patient No. | Mitotic count per 1000 cells* $M_1$ | Mitotic count per 1000 cells* $M_2$ | Mitotic rate $R$ per 1000 cells/h | Mitotic duration $D = M/R$ (h) | Potential doubling time $T_P = \ln 2/R$ (h) | Cell loss rate $L = R - r$ per 1000 cells/h | Cell loss factor $\lambda = L/R$ |
|---|---|---|---|---|---|---|---|
| Growing | | | | | | | |
| 1 | 5·4 | 13·0 | 3·3 | 1·7 | 213·3 | 2·2 | 0·7 |
| 2 | 13·2 | 22·4 | 5·6 | 2·4 | 123·8 | 5·5 | 1·0 |
| 3 | 7·0 | 30·8 | 7·7 | 0·9 | 90·0 | 5·0 | 0·7 |
| Mean | 8·5 | 22·1 | 5·5 | 1·6 | 142·4 | 4·2 | 0·8 |
| (s.d.) | (4·1) | (8·9) | (2·2) | (0·7) | (63·7) | (1·8) | (0·2) |
| Decreasing | | | | | | | |
| 4 | 14·6 | 20·2 | 5·1 | 2·9 | 137·3 | 5·1 | 1·0 |
| 5* | 2·0 | 12·0 | 3·7 | 0·5 | 187·7 | 4·8 | 1·3 |
| 6 | 7·0 | 10·0 | 2·5 | 2·8 | 277·3 | 2·9 | 1·2 |
| Mean | 7·9 | 14·1 | 3·8 | 2·1 | 200·8 | 4·3 | 1·2 |
| (s.d.) | (6·4) | (5·4) | (1·3) | (1·4) | (70·9) | (1·2) | (0·1) |
| Static 9 | 4·0 | 6·0 | 1·5 | 2·7 | 462·1 | 1·5 | 1·0 |
| Overall Mean | 7·6 | 16·3 | 4·2 | 2·0 | 213·1 | 3·8 | 1·0 |
| (s.d.) | (4·7) | (8·5) | (2·1) | (1·0) | (126·3) | (1·6) | (0·2) |

* The interval between biopsies was 4 h, except for patient No. 5 in which it was 3·25 h.

TABLE III.—*Mitotic Counts and Calculated Potential Doubling Times for the 6 Florid Tumours*

| Patient No. | Mitotic count per 1000 cells* $M_1$ | Mitotic count per 1000 cells* $M_2$ | Mitotic rate $R$ per 1000 cells/h | Mitotic duration $D = M/R$ (h) | Potential doubling time $T_P = \ln 2/R$ (h) | Cell loss rate $L = R - r$ per 1000 cells/h | Cell loss factor $\lambda = L/R$ |
|---|---|---|---|---|---|---|---|
| 1 | 15·2 | 18·6 | 4·7 | 3·3 | 149·1 | 4·0 | 0·9 |
| 3 | 21·8 | 23·4 | 5·9 | 3·7 | 118·5 | 4·7 | 0·8 |
| 4 | 14·0 | 31·4 | 7·9 | 1·8 | 88·3 | 7·5 | 1·0 |
| 5* | 7·0 | 17·4 | 5·4 | 1·3 | 129·5 | 3·0 | 0·6 |
| 7 | 11·4 | 23·0 | 5·8 | 2·0 | 120·1 | 5·6 | 1·0 |
| 8 | 6·6 | 17·0 | 4·3 | 1·6 | 163·1 | 2·3 | 0·5 |
| Mean | 12·7 | 23·3 | 5·6 | 2·3 | 128·2 | 4·5 | 0·8 |
| (s.d.) | (5·7) | (6·9) | (1·3) | (1·0) | (26·1) | (1·9) | (1·9) |

* The interval between biopsies was 4 h, except for Patient No. 5 in whom it was 3·25 h.

TABLE IV.—*A Comparison of Nodular and Florid Tumours Seen Together in Each of 4 Patients*

| Patient No. | Nodular tumours: Histology | Nodular tumours: Actual doubling time $T_D$ (days) | Nodular tumours: Mitotic rate $R$ per 1000 cells/h | Florid tumours: Histology | Florid tumours: Actual doubling time $T_D$ (days) | Florid tumours: Mitotic rate $R$ per 1000 cells/h |
|---|---|---|---|---|---|---|
| 1 | Mixed | 27·1 | 3·3 | Mixed | 44·0 | 4·7 |
| 3 | Mixed | 10·7 | 7·7 | Anaplastic | 25·6 | 5·9 |
| 4 | Monocellular | −474·4 | 5·1 | Monocellular | 79·6 | 7·9 |
| 5 | Anaplastic | −27·0 | 3·7 | Anaplastic | 12·1 | 5·4 |

florid tumours. The florid tumour from Patient No. 3 was necrotic, and showed only a small effect of Colcemid, but the measurements are included in the results. The co-existing nodular and florid tumours were unusual in having similar histopathology in all but one patient (Table IV). With the exception of these 4 patients, however, nodular tumours had a mixed cell histological pattern and the florid tumours were monocellular, with sheaves of spindle cells predominating. Delayed hypersensitivity responses were estimated to a variety of synthetic antigens, and to autologous tumour extract, in all but Patients 9, 2 and 6. Florid lesions were used as a source of tumour cells in those patients with that form of the disease. The results showed negative responses to tumour extract in all patients, and generally impaired responses to synthetic antigens in those with florid lesions (Taylor *et al.*, 1971*b*).

*Actual growth rate*

There was considerable spread in the actual doubling times of growing tumours, the longest being in Patient No. 2, who had nodules alone. The growing nodules had a range of doubling times from 10·65 to 202·55 days (mean 80 $\pm$ 106) whilst halving times for regressing nodules varied between 27 and 474 days (mean 193 $\pm$ 245).

The range of the actual clinical doubling times for the florid tumours was from 12·09 to 172·10 days, with a mean of 58 $\pm$ 61 days. There is no statistically significant difference between the actual clinical doubling times of the growing nodular tumours (80 days) and the florid tumours (58 days), but if the whole group of nodular tumours, including the static and the decreasing tumours, are considered, our tumour measurements may be said to confirm earlier reports (Kyalwasi, 1969; Taylor *et al.*, 1971*a*), of a difference in growth rate between nodular and florid tumours, the former having the longer doubling time.

*Cellular kinetics*

Spindle cells formed the greater part of all tumours, blood vessels and fibrous strands being rare, but necrotic areas were seen in florid tumours. The cells adjacent to vascular slits appeared to divide more frequently than others. The appearance after Colcemid confirmed earlier reports (Clarke, 1971), but anaphases and telophases were not seen.

The mitotic count was very variable, with a slight tendency to lower values for the nodular than for the florid tumours. The differences (7·6 against 12·7 mitoses per 1000 cells) is not statistically significant using Student's *t* test ($0{\cdot}2 > P > 0{\cdot}1$). The florid tumour of Patient No. 1 had a mixed cell pattern but a high mitotic count. This was reported as unusual by the pathologist making the initial diagnosis. There was no difference between the rates of cell production in the two forms of tumour, which were 4·20 and 5·62 per 1000 cells/h in nodular and florid tumours respectively.

The calculated mitotic duration varied widely, from 0·54 h in a decreasing tumour to 3·73 h in a florid tumour, and it was therefore not possible to correlate the mean figures with tumour morphology. The potential doubling times also varied considerably, from 90 h in a growing nodular tumour to 462 h in the single static tumour. The great variation forbids any comparison of means.

The cell loss factor was similar for florid tumours and growing nodular tumours, but significantly higher for decreasing nodular tumours ($0{\cdot}01 > P > 0{\cdot}001$, Student's *t* test). The regression of nodular tumours is due to a combination of a normal rate of cell production and a very high rate of cell loss. Thus, at the time of observation, these tumours lost each hour 30% more of their spindle cells than were formed by division. The slight tendency to a more rapid clinical growth in the florid tumours than in the growing nodular tumours may be a consequence of both a slightly higher mitotic rate and a slightly lower cell loss factor in the increasing nodular tumours than in the florid tumours. However, these differences are not statistically significant.

### DISCUSSION

The measurement of the parameters of the cell population kinetics in clinical tumours is, as mentioned, always difficult. Our results are based on relatively few observations made with methods of moderate exactness. The results should therefore be accepted only as provisional, and certainly not as exact values. This is especially so for the calculated averages within each group.

Tumour measurements in patients with Kaposi's sarcoma show that, when growing, this tumour behaves like many other human malignancies. The clinical doubling time of growing nodular and florid tumours varied from 11 to 203 days, 1 tumour was static while under observation, and 3 tumours regressed, with halving times varying from 27 to 474 days. In fibrosarcomas the clinical doubling time varies from 32 to 275 days (Breur, 1966).

Comparisons have been made between Kaposi's sarcomata and lymphomata, particularly Hodgkin's disease (Lukes and Butler, 1966), and Burkitt's lymphoma (McKinney, 1967). The growth rate of Kaposi's sarcoma is similar to that of Hodgkin's disease (Charbit, Malaise and Tubiana, 1971), but much slower than Burkitt's lymphoma, which has a doubling time of 3 days (Iversen *et al.*, 1974).

The calculation of cell production rate is based on arrest by Colcemid of the dividing cells in metaphase. Tannock (1967) doubted that colchicine was an ideal stathmokinetic drug, but Nome (1975) showed that this objection is not valid for Colcemid when a correct interval between injection and measurement is chosen. There is evidence in the litera-that stathmokinetic methods may be used favourably to study tumour kinetics over a 4-h period (Iversen, 1967; Refsum and Berdal, 1967; Smith, Thomas and Riches, 1974). Anaphases and telophases were not seen in the sections from tumours exposed to Colcemid, and this leads us to believe that the block was complete, and supports previous observations in Burkitt's lymphoma (Iversen *et al.*, 1974). The small Colcemid effect in one of the florid tumours was probably partly due to impaired vascular supply, areas of necrosis being seen in the histological sections. The significance of areas of necrosis in kinetic calculations can be assessed using methods such as that reported by Smith *et al.* (1974).

The stathmokinetic method has been used to measure cell production in experimental tumours (Elgjo, 1966; Nome, 1975). Following an initial biopsy and injection of Colcemid, further biopsies were taken at 30-min intervals. A graph of arrested metaphases (Y axis) plotted against time (X axis) revealed that the line of best fit, when projected downwards, crossed the Y axis at a point close to zero. In calculating the mitotic rate of mouse epidermis Elgjo found that there was little difference between the result using the line of best fit, and that obtained by drawing a line from the mitotic count 4 h after Colcemid to the intercept of the X and Y axes. This finding may be explained by the observation that the mitotic count without a stathmokinetic substance, which comprises all mitotic phases, is almost always higher than the initial counts after Colcemid. Thus after the injection of a stathmokinetic substance the mitotic count regularly falls during 30 min and then rises again. At 1 h the number of metaphase arrests is similar to the count without stathmokinesis, and only increases thereafter.

The mitotic rate of human tumours would ideally be measured using the slope of the line of best fit through serial observations. However, this is impossible because patients cannot be exposed to a series of biopsies at 30-min intervals. In view of the work of Elgjo (1966) and

Nome (1975), we feel that under these circumstances the most accurate method of measuring the cell production is to use the slope of a straight line from the X and Y intercept through the mean value of the mitotic index at 4 h.

Measurement of the rate of cell production gave a mean of about 4–5 cells/1000 cells/h, with a range of 1·5 to 7·9. This shows that the cells of Kaposi's sarcomas multiply more slowly than the cells of many other human tumours. Studies reported in the literature (for review, see Iversen, 1976) show that there is wide variation in the proliferative activity of different types of malignant tumours, of different tumours of the same histological type, and even of different areas of histologically similar fields from the same tumour. When the cell production rate is used for calculation of the potential doubling time, the results for human malignant tumours given in the literature show variations from 2 to 100 days.

Calculations of the mitotic duration gave values around 2 h, with a range of 0·54 to 3·73. The mitotic duration in normal tissues is usually around 1 h. A prolonged mitotic duration has been observed in some tumours (Iversen, 1976), and a long mitotic duration probably contributes to the high number of mitoses seen in many tumours.

The cell loss factor in Kaposi's florid and growing nodular tumours (*viz.* 0·8) is similar to values reported from other human malignancies in the literature (for a review, see Iversen, 1976).

One of the tumours was not growing, and 3 were apparently regressing. In the case of Patient No. 4 the measured difference in diameter from the first to second occasion was only 0·2 mm and may have been due to local pressure by garments or bedclothes. However, these patients with regressing tumours were under observation in a ward between measurements and, as the epidermal covering was intact at the second measurement, we believe that the results reflect real loss of tumour tissue and not the result of local trauma. These 3 tumours had cell loss factors higher than 1·0, and such results have never before been reported in kinetic studies of human malignancies. It is also likely that the regressing tumours had a growth fraction of considerably less than 1·0. The cell proliferation rate is calculated as the rate of cells entering mitosis/1000 cells among all viable cells, and is thus not dependent upon knowledge of the growth fraction in these tumours. The fact that some tumours grew, while others regressed, is not due to technical difficulties in measurement, but is an obvious clinical observation, as shown in the examples in Figs. 1 and 2.

Regrettably, none of our patients had both a growing and a regressing nodule which we were able to measure (see Figs. 1 and 2). Unusually, in 3 patients with co-existent nodules and florid tumours, the 2 forms had the same histological pattern (Table IV). In the first (Patient No. 1), the nodule was atypical and exhibited faster growth and a higher proliferation rate than the florid lesion. In 2 patients (Nos. 4 and 5) nodular regression was associated with a low proliferation rate and high cell loss

The mean mitotic rate for nodules (4·2/1000 cells/h) was lower than that for florid lesions (5·6) and the potential doubling time was correspondingly increased, being 213 h for nodules and 128 h for the florid lesions. These observations further characterize the 2 clinical groups. However, there is also a higher rate of cell loss in the florid tumours, and it may be that a high growth fraction is partly responsible for the rapid increase in size seen in some of these lesions.

It is possible that the cells of the regressing nodules carried an antigen not present in the cells of the florid lesion, and that immune responses to that antigen were responsible for the regression. This appears unlikely, as the 2

forms of tumour had similar histology. Moreover, in those patients with fungating tumours, delayed hypersensitivity responses were depressed to synthetic antigens and absent to an extract made from cells of the fungating tumour. Another explanation appears to be that some local factor was responsible for the regression. This might be physical pressure due to inelasticity of skin collagen in the more superficial tumours. Alternatively, it may be that there is in fact a smaller growth fraction of the cells in these regressing tumours, and that the other cells manufacture vasoactive substances such as those described by Bhana, Hillier and Karim (1971) which, acting locally, restrict or impair vascular perfusion. Local factors may be supplemented by cell-mediated immune mechanisms directed against tumour antigens in those patients where all the nodules show spontaneous regression.

The authors wish to thank Mrs V. Macmillan, Mr L. Sebwami and Mr M. Findlay for technical assistance. Professor A. Templeton reviewed the histological sections. We are grateful to Professor R. Owor, Professor S. Kyalwazi, Professor F. Deinhardt, and Dr M. A. Ball and Miss L. Marshall for additional assistance.

The work was supported by the Cancer Research Campaign, by Contract Nos. PH 43-67-1343 and PH 43-62-179, National Cancer Institute, National Institutes of Health, Bethesda, Maryland, the E. & M. Dawson Trust, Makerere University, and the Norwegian Aid for Developing Countries (NORAD).

### BEFERENCES

BHANA, D., HILLIER, K. & KARIM, S. M. N. (1971) Vasoactive Substances in Kaposi's Sarcoma. *Cancer, N.Y.*, **27**, 233.

BJERKNES, R. (1974) Exponential Growth. *Eur. J. Cancer*, **10**, 165.

BREUR, K. (1966) Growth Rate and Radiosensitivity of Human Tumours. *Eur. J. Cancer*, **2**, 157.

CHARBIT, A., MALAISE, E. P. & TUBIANA, M. (1971) Relation between the Pathological Nature and the Growth Rate of Human Tumours. *Eur. J. Cancer*, **7**, 307.

CLARKE, R. M. (1971) A Comparison of Metaphase Arresting Agents and Tritiated Thymidine Autoradiography in Measurement of Entry of Cells into Mitosis in the Crypts of Lieberkuhn. *Cell Tissue Kinet.*, **4**, 263.

ELGJO, K. (1966) Epidermal Cell Population Kinetics in Chemically Induced Hyperplasia. Thesis. Universitetsforlaget, Oslo.

IVERSEN, O. H. (1967) Kinetics of Cellular Proliferation and Cell Loss in Human Carcinomas. *Eur. J. Cancer*, **3**, 389.

IVERSEN, O. H. (1976) Kinetic Characterization of Malignant Tumours. In: *Proc. VI Int. Symp. Biological Characterization of Human Tumours.* Amsterdam: Excerpta Medica. p. 173.

IVERSEN, O. H., IVERSEN, U., ZIEGLER, J. L. & BLUMING, A. Z. (1974) Cell Kinetics in Burkitt Lymphoma. *Eur. J. Cancer*, **10**, 155.

KAPOSI, M. (1872) Idiopathisches Multiples Pigmentsarkom der Haut. *Arch. Dermat. Syph.*, **4**, 265.

KYALWASI, S. K. (1969) Treatment of Kaposi's Sarcoma. *E. Afr. med. J.*, **46**, 450.

LOTHE, F. (1963) Kaposi's Sarcoma in Ugandan Africans. *Acta path. microbiol. Scand.*, Suppl. **161**, 1.

LUKES, R. J. & BUTLER, J. J. (1966) The Pathology and Nomenclature of Hodgkin's Disease. *Cancer Res.*, **26**, 1063.

McKINNEY, B. (1967) Kaposi's Sarcoma and Burkitt Lymphoma. *E. Afr. med. J.*, **44**, 417.

NOME, O. (1975) Demecolcin and Vinblastine Sulphate as Stathmokinetic Agents for Different Tissues of the Hairless Mouse. *Path. Eur.*, **10**, 221.

REFSUM, S. B. & BERDAL, P. (1967) Cell Loss in Malignant Tumours in Man. *Eur. J. Cancer*, **3**, 235.

SMITH, S. R., THOMAS, D. B. & RICHES, A. C. (1974) Cell Production in Tumour Isografts Measured using Vincristine and Colcemid. *Cell Tissue Kinet.*, **7**, 529.

TANNOCK, I. F. (1967) A Comparison of the Relative Efficiencies of Various Metaphase Arrest Agents. *Expl Cell Res.*, **47**, 345.

TAYLOR, J. F., TEMPLETON, A. C., VOGEL, C. L., ZIEGLER, J. L. & KYALWAZI, S. K. (1971*a*) Kaposi's Sarcoma in Uganda: A Clinico-pathological Study. *Int. J. Cancer*, **8**, 122.

TAYLOR, J. F., JUNGE, U., WOLFE, L., DEINHARDT, F. & KYALWAZI, S. K. (1971*b*) Lymphocyte Transformation in Patients with Kaposi's Sarcoma. *Int. J. Cancer*, **8**, 468.

TAYLOR, J. F. & ZIEGLER, J. L. (1974) Delayed Cutaneous Hypersensitivity Reactions in Patients with Kaposi's Sarcoma. *Br. J. Cancer*, **30**, 312.

VOGEL, C. L., TEMPLETON, C. J., TEMPLETON, A. C., TAYLOR, J. F. & KYALWAZI, S. K. (1971) Treatment of Kaposi's Sarcoma with Actinomycin D and Cyclophosphamide. *Int. J. Cancer*, **8**, 136.